# Resveratrol promotes sensitization to Doxorubicin by inhibiting epithelial‐mesenchymal transition and modulating SIRT1/β‐catenin signaling pathway in breast cancer

**DOI:** 10.1002/cam4.1993

**Published:** 2019-01-29

**Authors:** Xiaoxia Jin, Yingze Wei, Yushan Liu, Xiaoyun Lu, Fei Ding, Jiatai Wang, Shuyun Yang

**Affiliations:** ^1^ Department of Pathology Nantong Tumor Hospital Nantong China

**Keywords:** breast cancer, Doxorubicin, Drug resistance, EMT, resveratrol, SIRT1, β‐catenin

## Abstract

Breast cancer is one of the leading fatal diseases for women worldwide who cannot have surgery typically have to rely on systemic chemotherapy to extend their survival. Doxorubicin (DOX) is one of the most commonly used chemotherapeutic agents against breast cancer, but acquired resistance to DOX can seriously impede the efficacy of chemotherapy, leading to poor prognosis and recurrences of cancer. Resveratrol (RES) is a phytoalexin with pharmacological antitumor properties, but its underlying mechanisms are not clearly understood in the treatment of DOX‐resistant breast cancer. We used cell viability assays, cell scratch tests, and transwell assays combined with Western blotting and immunofluorescent staining to evaluate the effects of RES on chemoresistance and the epithelial‐mesenchymal transitions (EMTs) in adriamycin‐resistant MCF7/ADR breast cancer cells, and to investigate its underlying mechanisms. The results showed that a treatment of RES combining with DOX effectively inhibited cell growth, suppressed cell migration, and promoted cell apoptosis. RES reversed EMT properties of MCF7/ADR cells by modulating the connection between SIRT1 and β‐catenin, which provides a hopeful therapeutic avenue to conquer DOX‐resistance and thereby prolong survival rates in breast cancer patients.

## 1 INTRODUCTION

The incidence of breast cancer has increased rapidly in recent years, making it become the most common malignancy in females worldwide with about 278,000 new cases and 64,000 deaths in 2013.^1^ The occurrence of breast cancer is usually associated with endocrine factors, genetic mutation, procreation, and precancerous lesions. Therapeutic methods for breast cancer include surgery excision, endocrine therapy, radiotherapy, and/or chemotherapy, with approximately 40% of patients receiving chemotherapy.^2^, ^3^ Chemotherapy has been considered as an effective treatment for locally primary/advanced and metastatic breast cancers. Doxorubicin (DOX), as a clinically used chemotherapeutic drug, has a broad antineoplastic spectrum and effects on a variety of tumors.^4^ However, chemoresistance to DOX can impede the therapeutic effect, failing to prolong survival, can bring about undesirable effects for tumor patients.^5^ Thus, we tried to find a chemical sensitizing agent and a side effect attenuator within the chemotherapy regimen for breast cancer that could alleviate the DOX‐resistance in a versatile approach, and then to explore the molecular mechanism that produced this result as well.

Many phytochemicals have been found to suppress the growth of cancer cells without affecting normal cells, to exhibit chemoprophylaxis effects, and to promote the sensitivity of cancer cells to DOX.^6^ Resveratrol (RES), known as a type of polyphenol compounds extracted from peanut, grape (red wine), tiger cane, mulberry, and other plants, has attracted increasing attention because of its varied healthy benefits.^7^, ^8^ RES is of key importance in aging, neurological dysfunction, angiocardiopathy, and inflammation diseases. It was also recognized to have antitumor and chemoprevent activity in connection with the lung, gastric, prostate, and breast cancer.^9^, ^10^ Furthermore, RES was reported to have synergistic effect with DOX in gastric cancer cells and reverse multidrug resistance in acute myeloid cells.^11^, ^12^ Thus, combined treatment with DOX and RES might be a potentially promising chemotherapy approach for breast cancer, but the relevant mechanisms remain to be explored. Recent studies indicated that the occurrence and development of epithelial‐mesenchymal transition (EMT) might be the crucial factor in the cell migration and DOX‐resistance of cancers.^13^, ^14^ However, whether RES takes effect on the DOX‐resistance and metastasis of breast cancer cells remains to be further studied, and the transformation of EMT in this process should be investigated as well.

In the present study, adriamycin‐resistant MCF7/ADR breast cancer cells exhibited enhanced aggressive migratory characteristics and EMT phenotype. RES successfully alleviated cell migration and increased synergistic sensitivity to DOX through reducing EMT processes and regulating the correlation between silent mating type information regulation 2 homologue 1 (SIRT1) and β‐catenin. Our work suggests new avenues for use of RES as a potential effective adjuvant drug in breast cancer treatment to overcome DOX‐resistance as well as tumor metastasis. Further investigation of the potential mechanisms for RES use with DOX and in prospective clinical trials is warranted.

## 2 MATERIALS AND METHODS

### 2.1 Cell culture

Human breast cancer cell lines MDA‐MB‐231, MCF7, MCF7/ADR, and benign breast epithelial cell line MCF‐10A were obtained from Chinese Type Culture Collection (Shanghai, China). MDA‐MB‐231 cells were cultured in Dulbecco's modified eagle medium (DMEM, Gibco, Grand Island, NY, USA) supplemented with 10% fetal bovine serum (FBS, Gibco) at 37℃ humidified incubator with 5% CO_2_. MCF7 and MCF7/ADR cells were cultured in RPMI 1640 medium (Gibco) supplemented with 10% FBS at the same atmosphere mentioned above. MCF‐10A cell line was cultured in a 1:1 ratio of DMEM/F‐12 nutrient mixture (Gibco) medium supplemented with 10% FBS.

### 2.2 Antibodies and reagents

Antibodies including anti‐Vimentin, anti‐E‐cadherin, anti‐N‐cadherin, anti‐β‐catenin, and anti‐SIRT1 were purchased from Abcam (Cambridge, MA, USA); anti‐GAPDH was obtained from SantaCruz (CA, USA).

Doxorubicin and resveratrol used in this study were purchased from Sigma (St. Louis, MO, USA). DOX was scale‐diluted with phosphate buffered saline (PBS) buffer while RES was diluted in dimethylsulfoxide (DMSO) solvent, and then was stored at −80℃. MG132 and cycloheximide (CHX) were obtained from Selleck (Houston, TX, USA), both were diluted in DMSO solvent in a stock concentration.

### 2.3 Lentivirus and transfection

The negative SIRT1 small hairpin RNA (shSIRT1, 5’‐GGCTTGATGGT AATCAGTA‐3’, 5’‐TACTGATTACCATCAAGCC‐3’) were cloned into lentivirus vector (GV248) to form GV248‐shCon and GV248‐shSIRT1 lentivirus by Genechem (Shanghai, China).

Lentivirus infection was operated as protocol from Genechem described. Stable cell lines were generated in 24‐well plates with serum‐free medium. MCF7/ADR cells were transfected with shSIRT1 lentivirus at the infection MOI ≥ 90 for 24 hours. Then cells were cultured in the medium with 10% FBS and continuously cultured for another 6 days followed by selection with G418 (Invitrogen, Carlsbad CA, USA) at 500 μg/mL.

### 2.4 Cell Counting Kit‐8 assay

To detect cell proliferation ability, we used cell Counting Kit‐8 (CCK8, DOJINDO, Japan). First, about 1 × 10^4 ^cells contained in 100 μl medium were seeded in each well of 96‐well plates. After cultivation for 24 hours, cells were treated with or without different doses of DOX or/and RES at different time points, respectively. For investigation, cells were incubated with 10 μL/well CCK‐8 solution for 2 hours, then the number of viable cells were measured and calculated by the absorbance at 450 nm.

### 2.5 Cell scratch test

Cells were seeded into 6‐well plates and reached a confluence in 24 hours, then a 100 μL pipette tip was used to make a single scratch. After washed with PBS, cells were incubated with FBS‐free culture medium alone or containing DOX and/or RES. Olympus IX71 inverted microscope was used to capture images of the scratches at different time points (0, 24 and 48 hours).

### 2.6 Transwell migration assay

Cell migration was investigated by transwell migration assay using transwell inserts with an 8 μm pore filter (BD bioscience, SanJose, CA, USA). Cells were with or without DOX and/or RES for 48 hours and then trypsinizated to cell suspension. Next, serum‐free medium contained with 4 × 10^4 ^cells were seeded into the upper chamber while complete medium was added to the lower chamber. After incubation for 24 hours, the cells were fixed with paraformaldehyde for 15 minutes and stained with crystal violet (Beyotime Biotechnology, Beijing, China) for 10 minutes. Next, the cells on the upper surface of the membrane were wiped off, and cells on the lower membrane were surveyed by Leica microscope. Migration ability of cells was evaluated by the average number of migrated cells from four random fields.

### 2.7 Colony formation assay

After untreated or treated with DOX and/or RES for 48 hours, cells were seeded into each well of 6‐well culture plates with a density of 500 cells/well. After cultured in drug‐free culture for another 14 days, cells were fixed with paraformaldehyde for 15 minutes and stained with crystal violet for 10 minutes to visualize colonies. Only ≥50 cells regarded as positive colonies were counted and compared.

### 2.8 TUNEL assay

For TUNEL assay, the cells were washed twice with PBS and fixed with formaldehyde for 20 minutes, then were permeabilized with 0.1% TritonX‐100 for 10 minutes. After being washed in PBS for 5 minutes, cells were incubated with TdT reaction mixture at 37℃ in a dark humidified chamber for 60 minutes. Next, the cells were immersed in 2 × SSC buffer for 15 minutes to stop the reaction. After being washed with PBS, the cell nuclei were counterstained with DAPI (Beyotime Biotechnology). Finally, images were captured using a fluorescence microscope (TCS‐SP2, Leica, Germany).

### 2.9 Immunofluorescence technique

Cells were seeded in cofocal dishes and treated with the corresponding ways. Cells were fixed with paraformaldehyde for 30 minutes and then with 0.2% TritonX‐100 for 10 minutes. After being washed with PBS, cells were blocked by normal goat serum supplemented with 10% albumin from bovine serum for 30 minutes. Then cells were incubated with the primary antibodies including anti‐Vimentin (diluted to 1:500), E‐cadherin (diluted to 1:200), and anti‐β‐catenin (diluted to 1:50) overnight at 4℃. After being washed with PBS, cells were incubated with anti‐rabbit secondary antibody conjugated to Alexa‐488 fluorescence (Invitrogen) for 2 hours at the room temperature. At last, the nuclei were stained with DAPI for 15 minutes and intracellular localization of target proteins were observed with a fluorescence microscope.

### 2.10 Co‐immunoprecipitation assay

Antibodies against SIRT1 and β‐catenin were used to be incubated with protein A/G beads (ThermoFisher Scientific, MA, USA) for 6 hours, respectively. After being washed with TBS for three times, the antibodies conjugated beads were incubated with the cell lysates at 4℃ overnight. Then the beads were washed with TBS and centrifuged at 19 000 ***g*** for 5 minutes. After discarding the supernatant, the equivoluminal SDS buffer was added into the beads. Lastly, the beads were boiled for 5 minutes and the target proteins were detected by Western blotting.

### 2.11 Western blot analysis

Cultured cells were lysed in RIPA buffer (Beyotime Biotechnology) directly and the concentration was determined by BCA Protein Assay Kit (Beyotime Biotechnology). Proteins with the same concentration were segregated on SDS‐PAGE gels and transferred onto PVDF membranes (Millipore, Danvers, MA, USA). After blocked by 5% skim milk, the membrane was incubated with the primary antibodies at 4℃ overnight. The next day, the membrane was washed with TBS‐T buffer and then incubated with appropriate secondary antibodies at 37℃ for 2 hours. Finally, the samples were detected by the ECL system (ThermoFisher).

### 2.12 Statistical analysis

Data were expressed as means ± SD from at least three independent experiments. SPSS 19.0 software was used to perform statistical analysis. Student's t test was performed to evaluate the differences between individual groups. *P *values <0.05 were considered to be statistically significant and graphs were created with GraphPad Prism 5.0 software.

## 3 RESULTS

### 3.1 Effects of DOX and RES on breast cancer cells

**Figure 1:**
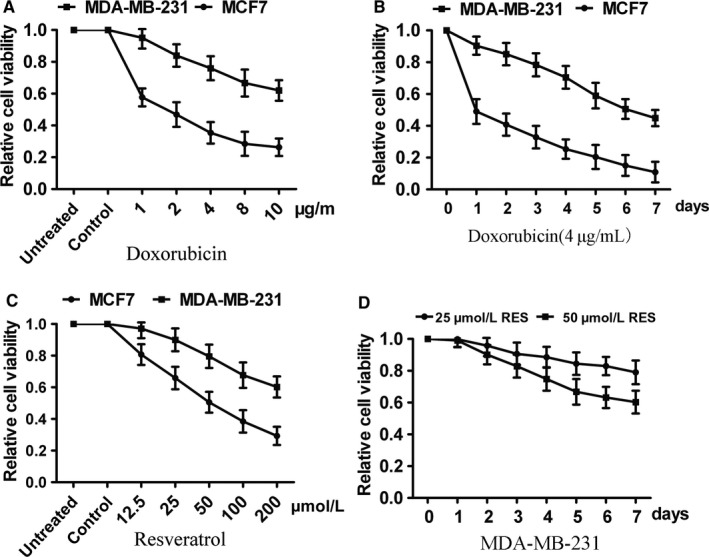
Effects of DOX and RES on breast cancer cells. (A) The chemo‐sensitivity of MCF7 and MDA‐MB‐231 cells to DOX treatment was detected by CCK8 assay. (B) The survival inhibition effect of 4 μg/mL DOX treated for 7 days on MCF7 and MDA‐MB‐231 cells was detected by CCK8 assay. (C) The survival inhibition effect of RES with the concentration from 0 to 200 μmol L^−1^ on MCF7 and MDA‐MB‐231 cells. (D) The survival inhibition effect of 25 and 50 mmol L^−1^ RES treated for 7 days on MDA‐MB‐231 cells

We detected the chemical sensitivity of MCF7 and MDA‐MB‐231 cells to DOX and RES treatment by CCK8 assay, respectively. Concentration gradient of DOX was from 0 to 10 μg/mL. The survival rate of MCF7 cells was inhibited by DOX, and the inhibition rate increased along with the increase in treatment time and concentration (Figure 1A). However, DOX did not inhibit the survival of MDA‐MB‐231 cells in a dose‐ and time‐dependent manner until its concentration reached 4 μg/mL. Besides this, survival rate of MDA‐MB‐231 cells was still as high as 45% after 7‐day treatment of 2 μg/mL DOX while MCF7 cells presented with 15% only (Figure 1B). Then cells were treated with RES with the concentration from 12.5 to 200 μmol L^−1^M. As the same, RES significantly inhibited cell survival of MCF7 cells in a dose‐ and time‐dependence manner whereas RES had no obviously suppression effect on MDA‐MB‐231 cells until its concentration exceeded 50 μmol L^−1^ (Figure 1C). As the previously found, the 7‐day survival rate of MDA‐MB‐231 cell maintained over 80% when treated with 25 μmol L^−1^ RES and about 60% in 50 μmol L^−1^ treatment (Figure 1D).

### 3.2 DOX‐resistant cells MCF7/ADR exhibited enhancive migratory phenotype

**Figure 2:**
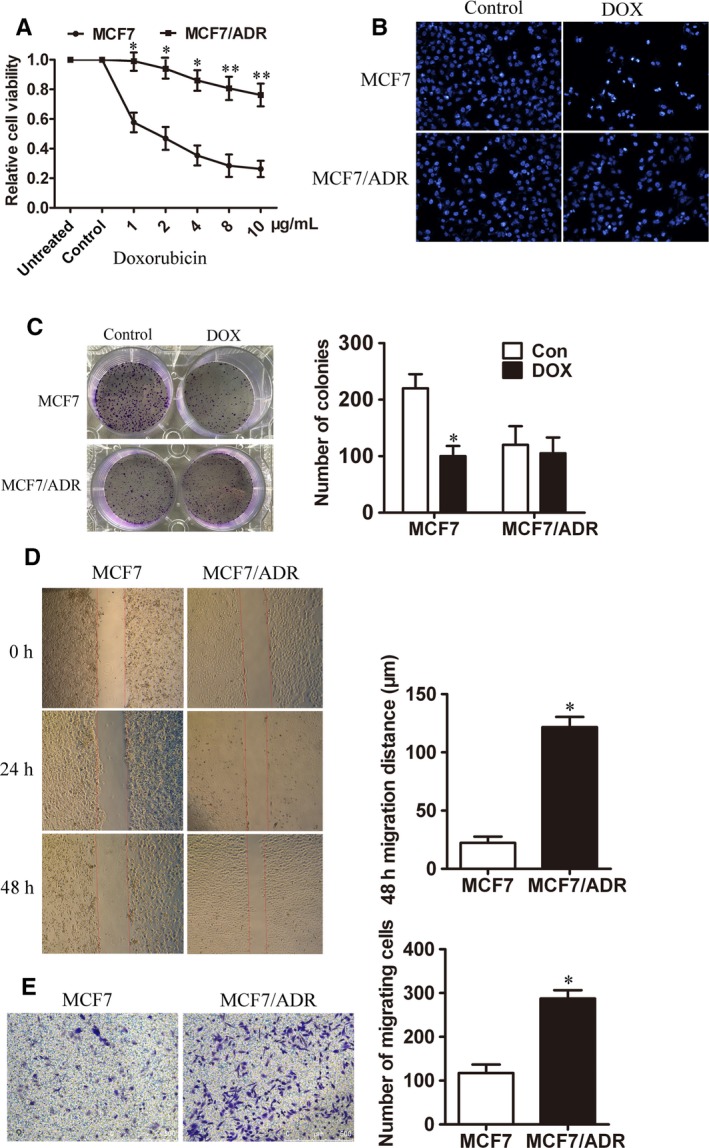
DOX‐resistant cells MCF7/ADR exhibited enhancive migratory phenotype. (A) The chemo‐sensitivity of MCF7 and MCF7/ADR cells to different concentrations of DOX for 48 h treatment was evaluated by CCK8 assay (n = 3, **P < 0.01, *P < 0.05). (B) The nuclei of MCF7 and MCF7/ADR cells were stained by DAPI after treated with 4 μg/mL DOX for 48 h. (C) Colony formation was performed to detect the growth of MCF7 and MCF7/ADR cells treated with 4 μg/mL DOX (n = 3, *P < 0.05). (D) The migration ability of MCF7 and MCF7/ADR cells was measured by cell scratch test (Bar = 750 μm, n = 3, *P < 0.05). (E) Transwell migration assay was used to detect the number of trans‐membrane cells (Bar = 500 μm, n = 3, *P < 0.05)

As both DOX and RES have obvious inhibitory effects on MCF7 cells, we selected MCF7 cells and MCF7/ADR cells as the suitable cell models to investigate the effects of RES on DOX‐resistance in breast cancer. CCK8 assay showed that MCF7/ADR cells had no significant change with the treatment of different concentrations of DOX while MCF7 cells had a visible decrease in cell vitality (Figure 2A). After being treated with low dose of DOX (4 μg/mL) for 48 hours, MCF7 and MCF7/ADR cell nuclei were stained by DAPI. It turned out that morphological changes including nuclear condensation and nuclear fragmentation happened on MCF7 cells while no changes occurred in MCF7/ADR cells (Figure 2B). Meanwhile, colony formation was performed to confirm that MCF7 cells had a slower growth compared with MCF7/ADR cells with the treatment of 4 μg/mL DOX (Figure 2C). These results suggested that MCF7/ADR cells maintained the resistant ability to DOX while MCF7 cells were sensitive to it. Next, we investigated the relation between DOX‐resistance characteristics of MCF7/ADR cells and its enhancive migratory phenotype. We detected cell migration ability by cell scratch test and transwell assay, and both results confirmed that the migration capacity of MCF7/ADR cells was greater than that of MCF7 cells (Figure 2D‐E).

### 3.3 MCF7/ADR cells showed significant EMT characteristics

**Figure 3:**
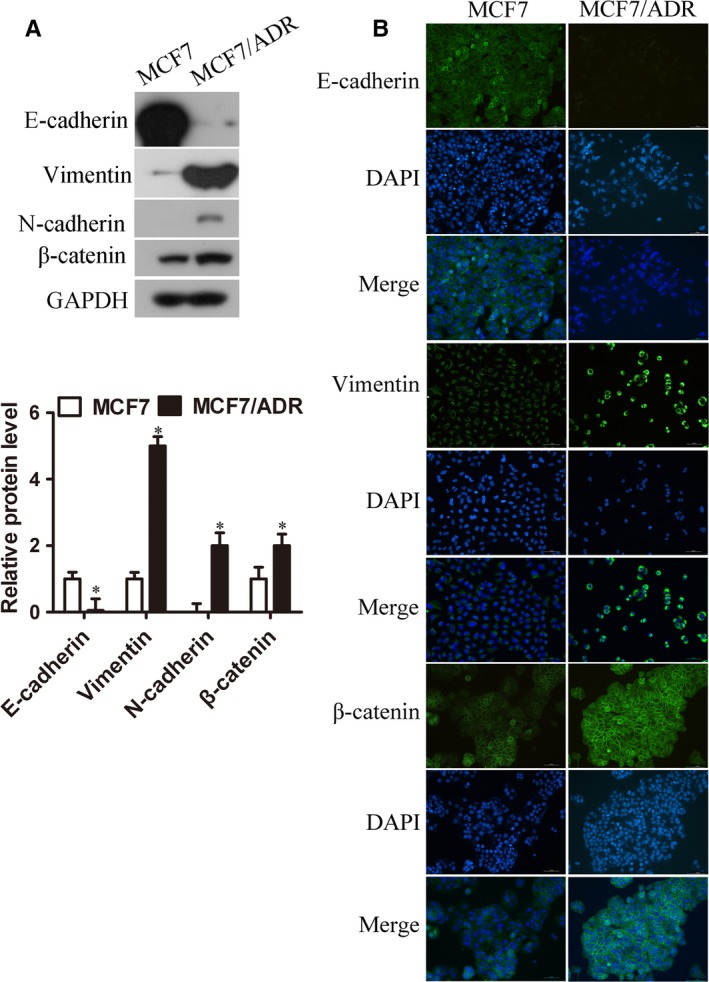
MCF7/ADR cells showed significant EMT characteristics. (A) Western blot was used to measure EMT‐related proteins from both MCF7 and MCF7/ADR cells (n = 3, *P < 0.05). (B) IF assay was used to detect EMT‐related proteins in both cells. E‐cadherin, Vimentin, and β‐catenin were all stained in green, and nuclei stained with DAPI were in blue (Bar = 70 μm)

As cell migration and drug resistance were related to EMT phenotype, we detected the EMT markers including E‐cadherin, N‐cadherin, Vimentin, and β‐catenin by Western blots (Figure 3A) and immunofluorescence (IF) (Figure 3B). The expression of epithelial‐specific marker E‐cadherin was lower in MCF7/ADR cells while mesenchymal markers N‐cadherin, Vimentin, and β‐catenin increased significantly compared with MCF7 cells, which indicated that MCF7/ADR cells presented with enhancive migration ability was possibly due to its transform in EMT phenotype.

### 3.4 RES combined with DOX effectively inhibited proliferation and migration of MCF7/ADR cells

**Figure 4:**
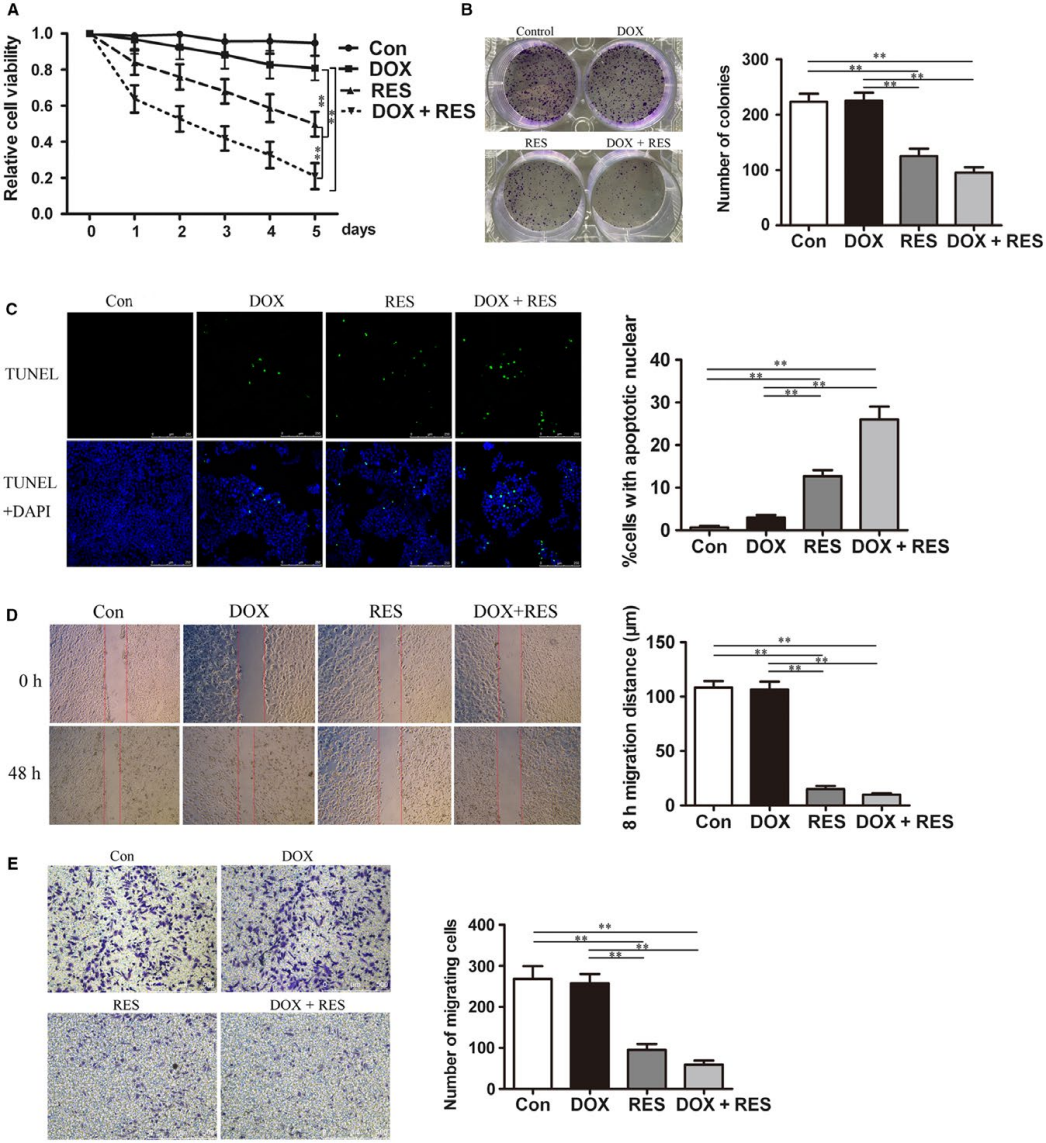
RES combined with DOX effectively inhibited proliferation and migration of MCF7/ADR cells. (A) MCF7/ADR cells were untreated or treated with 4 μg/mL DOX, 50 μmol L^−1^ RES, or both for 7 days, respectively and the cytotoxicity was detected by CCK8 assay (n = 3, **P < 0.01). (B) Colony forming ability of MCF7/ADR cells after being exposed to DOX or/and RES for 48 h was investigated by colony‐forming assay (n = 3, **P < 0.01). (C) MCF7/ADR cells were untreated or treated with DOX or/and RES for 48 h, followed by TUNEL analysis to detect cell apoptosis. The green fluorescence represented apoptotic bodies (n = 3, **P < 0.01). (D) The migration distance was measured to show the migration ability of MCF7/ADR cells after untreated or treated with DOX or/and RES for 48 h (Bar = 750 μm, n = 3, **P < 0.01). (E) MCF7/ADR cells were untreated or treated with DOX or/and RES for 48 h, followed by transwell migration assay was also used to detect cell migration (Bar = 500 μm, n = 3, **P < 0.01)

To investigate the effect of DOX combined with RES on the proliferation inhibition of MCF7/ADR cells, we treated cells with different concentration of DOX and RES for 48 hours. Compared with DOX or RES treated alone, the inhibitory effect in the combined group was significantly stronger than the single drug group. By calculating the combination index (CI) of each group, we found that the CI of each combination group was always less than 1, and the average value was 0.45, showing an obvious synergistic effect (Table 1). Thus, we treated cells with 4 μg/mL DOX or 50 μmol L^−1^ RES, either alone or in combination to detect the cytotoxicity of RES on MCF/ADR cells. Compared with untreated group, treatment with DOX (4 μg/mL) had no obvious effect on the cell proliferation via CCK8 assay. For the RES (50 μmol L^−1^)‐treated group, the inhibiting effect on cell viability showed a time dependence. Remarkably, treatment combined with DOX and RES showed the strongest inhibitory effect on MCF/ADR cells (Figure 4A). In addition, we analyzed whether combined treatment could suppress the colony formation of MCF/ADR cells. The results showed that combined treatment with DOX and RES had the strongest inhibitory effect on colony formation; also, RES treated alone had an inhibitory effect while DOX‐treated group had no effects on that (Figure 4B). The effect on cell apoptosis induced by DOX (4 μg/mL) and/or RES (50 μmol L^−1^) treatment was measured by TUNEL assay. It showed that there were no apoptotic cells observed in DOX‐treated alone group while RES treated alone could trigger cell apoptosis. Meanwhile, combined treatment with 4 μg/mL DOX and 50 μmol L^−1^ RES induced cell apoptosis maximally (Figure 4C). Next, the effect of RES on cell migration was detected by cell scratch test and transwell assay. In the same way, we treated cells with 4 μg/mL DOX or 50 μmol L^−1^ RES, either alone or in combination. Results of the cell scratch test suggested that DOX treatment alone had no significant effect on cell migration while RES decreased cell migration significantly in MCF7/ADR cells. Combination of DOX and RES treatment achieved a remarkably stronger migration‐inhibitory effect than RES or DOX treated alone (Figure 4D). Transwell assay also showed DOX treatment alone did not influence the transmembrane ability of MCF7/ADR cells whereas RES treated alone or combined with DOX, significantly decreased cell number that migrated through the membrane (Figure 4E). To sum up, combined treatment with DOX and RES effectively inhibited cell proliferation, promoted cell apoptosis, and suppressed cell migration of MCF7/ADR cells.

**Table 1:** Combination index (CI) values of DOX combined with RES in MCF7/ADR cells

DOX (μg/mL)+RES (μmol L^−1^)	Fa/%	CI
1 + 12.5	29.05	0.68
2 + 25	41.52	0.54
4 + 50	58.37	0.37
8 + 100	72.52	0.42
10 + 200	85.6	0.33

Fa represents as cellular proliferation inhibition rate.

The combination index (CI) defining the interaction between DOX and RES were analyzed by using CompuSyn software.

### 3.5 RES reversed EMT phenotype of MCF7/ADR cells and promoted the expression of SIRT1

**Figure 5:**
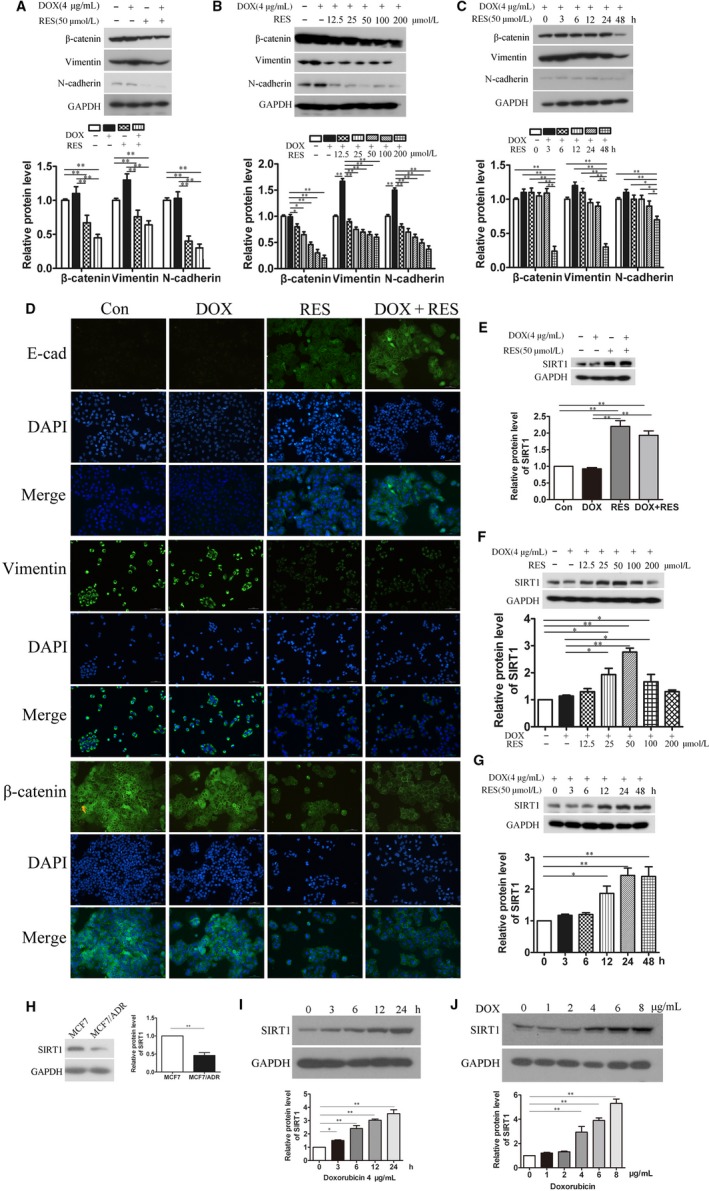
RES reversed EMT phenotype of MCF7/ADR cells and increased the expression of SIRT1. MCF7/ADR cells were treated with or without 4 μg/mL DOX, 50 μmol L^−1^ RES or both for 48 h. (A) Cells were harvested for Western blot analysis to detect the expression of EMT‐related proteins after treated for 48 h (n = 3, **P < 0.01). (B) MCF7/ADR cells were treated with or without 4 μg/mL DOX or/and RES of the concentration from 0 to 200 μmol L^−1^. Cells were harvested for Western blot analysis to detect EMT‐related proteins (n = 3, *P < 0.05, **P < 0.01). (C) MCF7/ADR cells were treated with or without 4 μg/mL DOX or/and 50 μmol L^−1^ RES, then cells were harvested at different time points (0, 3, 6, 12, 24, 48 h) for Western blot analysis to detect EMT‐related proteins (n = 3, *P < 0.05, **P < 0.01). (D) EMT‐related proteins were detected by IF assay. E‐cadherin, Vimentin, and β‐catenin were stained in green and nuclei were stained with DAPI in blue (Bar = 70 μm). (E) MCF7/ADR cells were treated with or without 4 μg/mL DOX, 50 μmol L^−1^ RES or both for 48 h. (F) MCF7/ADR cells were treated with or without 4 μg/mL DOX or/and RES with the concentration from 0 to 200 μmol L^−1^. (G) MCF7/ADR cells were treated with or without 4 μg/mL DOX or/and 50 μmol L^−1^ RES. Cells were harvested at different time points (0, 3, 6, 12, 24, 48 h). Western blot analysis was used to detect the expression of SIRT1 (n = 3, *P < 0.05, **P < 0.01). (H) Protein level of SIRT1 was determined by Western blot analysis in MCF7 and MCF7/ADR cells (n = 3, **P < 0.01). (I) Protein levels of SIRT1 in MCF7 cells were detected by Western blot analysis after being treated with 4 μg/mL doxorubicin at different time points (0, 3, 6, 12, 24 h) (n = 3, *P < 0.05, **P < 0.01). (J) Protein levels of SIRT1 in MCF7 cells were detected after being treated with doxorubicin at the different concentration (0, 2, 4, 6, 8, 10 μg/mL) for 24 h (n = 3, **P < 0.01)

Previous results indicated that MCF7/ADR cells showed enhanced migratory ability associated with EMT phenotype. Thus, we explored whether the effect of RES on MCF7/ADR cells was through transforming EMT phenotype. We treated MCF7/ADR cells with or without 4 μg/mL DOX or/and 50 μmol L^−1^ RES, then we investigated the expressions of EMT‐related proteins by Western blot and IF technology. Increased expressions of Vimentin, N‐cadherin, and β‐catenin were observed in DOX‐treated alone group while the treatment with RES alone decreased the expressions of Vimentin, N‐cadherin, and β‐catenin significantly compared to untreatment. Notably, in DOX and RES combined treatment group, RES antagonized DOX‐induced upregulation of Vimentin and N‐cadherin as well as β‐catenin via Western blots (Figure 5A). Furthermore, RES decreased expressions of Vimentin, N‐cadherin, and β‐catenin in a concentration‐ and time‐dependent manner (Figure 5B‐C). Similar findings on expressions of Vimentin and β‐catenin were observed by IF staining, and expression of E‐cadherin was upregulated significantly in RES‐treated group (Figure 5D). These findings revealed that RES possibly changed the expressions of EMT‐related molecules to resist DOX‐induced EMT, leading to prohibit the migration ability and the growth of MCF7/ADR cells. Thus, to clarify in which way RES modulated DOX‐resistance and enhanced EMT phenotype in MCF7/ADR cells was necessary. Because RES has been reported as the activator of SIRT1, we detected the expression of SIRT1 by Western blots. The result showed that RES induced an evident increased expression of SIRT1 (Figure 5E), which was also upregulated along with the increase in concentration and time (Figure 5F‐G). The protein level of SIRT1 in MCF7/ADR cells was lower than that in MCF7 cells was detected (Figure 5H). When MCF7 cells were treated with DOX of different concentration gradients (0, 2, 4, 6, 8, 10 μg/mL) at different time points (0, 3, 6, 12, 24 hours), the protein level of SIRT1 was always upregulated (Figure 5I‐J), reminding us of SIRT1’s inhibitory effect on DOX‐resistance. To determine the SIRT1’s role in RES‐mediated inhibiting effects on MCF7/ADR cells, we generated MCF7/ADR‐shSIRT1 cell line using shSIRT1 lentivirus to test cell proliferation, cell apoptosis, and cell migration after being treated with DOX and RES. Our results showed that shSIRT1 treatment significantly reversed inhibiting effects of RES on MCF7/ADR cells (Figure S2).

### 3.6 RES regulated SIRT1/β‐catenin signaling pathway in MCF7/ADR cells

**Figure 6:**
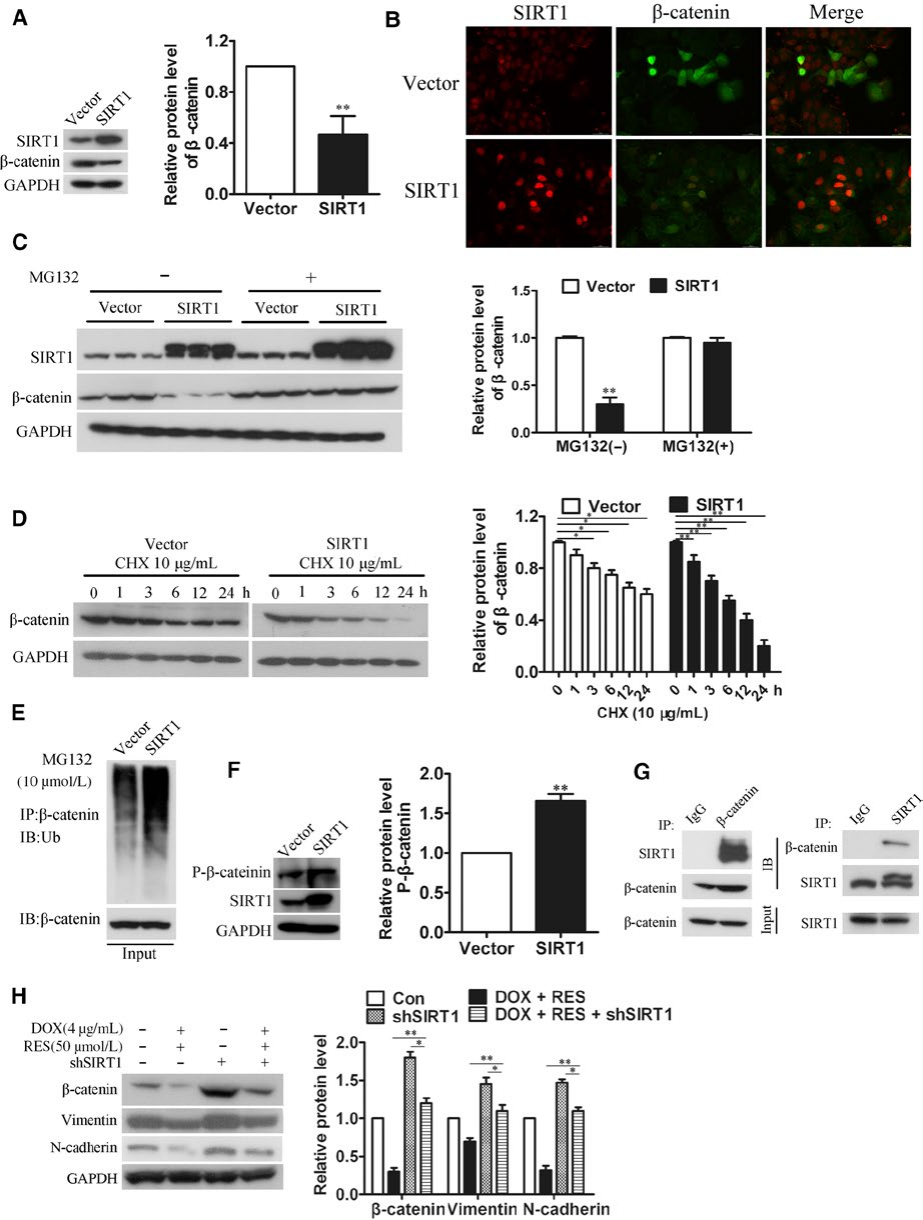
RES regulated SIRT1/β‐catenin signaling pathway in MCF7/ADR cells. (A) MCF7/ADR cells overexpressed with SIRT1 were harvested for Western blot analysis for β‐catenin (n = 3, **P < 0.01). (B) IF assay was used to detect the expression of β‐catenin in MCF7/ADR cells which overexpressed with or without SIRT1, β‐catenin were stained in green and SIRT1 was stained in red (Bar = 40 μm). (C) MCF7/ADR cells were transfected with SIRT1, then treated with 10 μmol L^−1^ MG132 for 6 h. Cells were harvested for Western blot analysis of β‐catenin (Vector: control cells without the overexpression of SIRT1, n = 3, **P < 0.01). (D) MCF7/ADR cells were transfected with SIRT1, then treated with 10 μg/mL CHX for different time (0, 1, 3, 6, 12, 24 h). Cells were harvested for Western blot analysis of β‐catenin (Vector: control cells without overexpressing SIRT1, n = 3, *P < 0.05, **P < 0.01). (E) MCF7/ADR cells were transfected with SIRT1, then treated with 10 μmol L^−1^ MG132 for 6 h. Cells were harvested and Western blot analysis was performed to detect ubiquitination level of β‐catenin (Vector: control cells without overexpressing SIRT1). (F) MCF7/ADR cells were transfected with SIRT1, then Western blot analysis was performed to analyze the phosphorylation of β‐catenin at Ser33/37/Thr417 sites (Vector: control cells without overexpressing SIRT1, n = 3, **P < 0.01). (G) Co‐IP assay indicated an interaction between SIRT1 and β‐catenin. Protein A/G beads were incubated with β‐catenin/SIRT1 for 6 h, respectively, followed by the incubation with cell lysate overnight. Then Western blot analysis was performed to detect expression of SIRT1 and β‐catenin, respectively. (H) MCF7/ADR or MCF7/ADR‐shSIRT1 cells were treated with 4 μg/mL DOX and 50 μmol L^−1^ RES, then the expressions of EMT‐related proteins including Vimentin, N‐cadherin, and β‐catenin were investigated by Western blot analysis (n = 3, *P < 0.05, **P < 0.01)

To illustrate how RES reverses EMT, we focused on β‐catenin which occupied a decisive position in proliferation, apoptosis, and migration of tumor cells. Previous results from Western blots showed that RES decreased the expression of β‐catenin, and promoted SIRT1 expression significantly. We sought to explore further whether SIRT1 modulated the expression of β‐catenin in RES‐treated cells. To do this, we detected decreased expression of β‐catenin in SIRT1‐overexpressed MCF7/ADR cells by Western blots and IF staining (Figure 6A‐B), which implicated an inverse relationship between the expressions of SIRT1 and β‐catenin. To elucidate the mechanism by which SIRT1‐repressed expression of β‐catenin, we sought to test whether SIRT1 could affect the stability of β‐catenin protein. It was known that β‐catenin was degraded through ubiquitin‐mediated proteolysis in various cancers. Thus, we detected the role of SIRT1 on the degradation and ubiquitination of β‐catenin in MCF7/ADR cells. We found that the protein level of β‐catenin was restored when SIRT1‐overexpressed MCF7/ADR cells were treated with proteasome inhibitor MG132 (Figure 6C). In addition, the half‐life of β‐catenin protein was significantly reduced in SIRT1‐overexpressed MCF7/ADR cells compared with the empty vector group (Figure 6D). These results similarly indicated that SIRT1 might inhibit the protein level of β‐catenin through ubiquitin‐mediated proteolysis. Indeed, the ubiquitination level of β‐catenin protein was increased in SIRT1‐overexpressed MCF7/ADR cells (Figure 6E). It has been reported that phosphorylation of β‐catenin was required for its ubiquitination and degradation by proteasome. Thus, we analyzed the phosphorylation of β‐catenin when SIRT1 was overexpressed in MCF7/ADR cells, and observed a marked increase (Figure 6F). In addition, Co‐IP assay was performed and indicated an interaction between SIRT1 and β‐catenin (Figure 6G). These data together demonstrated that SIRT1 induced β‐catenin degradation by promoting ubiquitin‐mediated proteolysis in MCF7/ADR cells. At the same time, we used MCF7/ADR‐shSIRT1 cell line to confirm the important role of SIRT1/β‐catenin pathway in RES‐treated cells. We treated MCF7/ADR or MCF7/ADR‐shSIRT1 cells with 4 μg/mL DOX and 50 μmol L^−1^ RES, then we detected the expressions of EMT‐related proteins by Western blot. Being treated with shSIRT1 antagonized RES‐induced deregulation of Vimentin and N‐cadherin as well as β‐catenin (Figure 6H), which showed that RES reversed EMT in a SIRT1/β‐catenin pathway dependent manner.

## 4 DISCUSSION

In the present study, we demonstrated for the first time that RES reversed DOX‐resistance, inhibited migration capacity of breast cancer DOX‐resistant cells by modulating EMT phenotype and SIRT1/β‐catenin pathway. We have shown that RES upregulated the expression of SIRT1, consequently suppressed, and thereby leading to the destruction of β‐catenin. We showed a new mechanism that underlies the progression of β‐catenin, and suggested that the molecular principle of RES may be used in DOX‐resistance in breast cancer.

For patients with advanced and metastatic cancers who have no opportunities to operate, chemotherapy is considered as the most efficient therapy which alleviates symptoms and improves survival rate. DOX is a commonly used chemotherapeutic drug which has been queried for severe side effects and the acquired drug resistance, as well as the development of EMT.^15^, ^16^ EMT is a biological transformation of epithelial cells to mesenchymal cells through which cells lose cell polarity and connections so that more migratory and invasive properties can be obtained.^17^ EMT is an important pathological process for the migration and invasion of tumor cells derived from epithelial cells.^18^ It not only promotes metastasis of cancer cells but also enhances the development of DOX‐resistance.^19^ EMT was reported to induce the overexpression of chemoresistance‐related genes and thus led to multidrug resistance in breast cancer.^20^, ^21^ DOX‐induced EMT also was detected in gastric cancer cells which was inhibited along with the suppression of β‐catenin signaling pathway.^22^ In addition, selective inhibitor of β‐catenin effectively inhibited EMT progress and enhanced chemo‐sensitivity of HER‐2 positive gastric cancer cells to lapatinib which targeted to HER‐2.^23^ A recent report has suggested that under the treatment of cyclophosphamide, the primary tumor cells showed EMT phenotype and chemoresistance exhibited by reduced cell proliferation and increased expression of multidrug resistant genes in prostate cancer.^24^ All these data indicated that EMT occupies an important position in chemoresistance and promotes metastasis in cancers. In this study, MCF7/ADR cells were confirmed to acquire DOX‐resistance and enhanced migration ability which showed in Figure 2. At the same time, higher expressions of mesenchymal cell markers such as β‐catenin, N‐cadherin, and Vimentin, as well as lower expression of epithelial cell molecule E‐cadherin were observed in MCF7/ADR cells compared with MCF7 cells (Figure 3).

To overcome DOX‐resistance of breast cancer cells and alleviate its adverse effects, we took RES, which is an eminent phytochemical with various health benefits, to collaborative treatment with DOX. In gastric cancer MGC803 cells, RES served as a chemosensitizer to DOX and inhibited cell cycle progression by targeting PTEN which was known as a negative regulator of PI3K/Akt pathway. Also PTEN has been regarded to reverse drug resistance mediated by EMT.^11^ In breast cancer, RES was reported to inhibit cell proliferation and increased cellular influx of DOX.^25^ Consistent with the previous study, our data revealed that RES effectively inhibit cell proliferation and promote cell apoptosis in MCF7/ADR cells. What's more, RES suppressed the growth of breast cancer cells without affecting mammary epithelial cells MCF10A (Figure S1). Moreover, we discovered that RES could suppress cell migration and transform the expression of EMT‐related proteins, suggesting that RES had an inhibitory effect to EMT on MCF7/ADR cells (Figure 4).

Resistance to DOX mediated by EMT in cancers is regulated by various signaling pathways, among which β‐catenin signaling gets our attention. β‐catenin was reported to regulate EMT‐related proteins and promote the expression and activity of ZEB1, which then repair DNA damage and reverse DOX‐resistance.^26^ In the present study, β‐catenin was found to high expressed in MCF7/ADR cells which confirmed its important roles in DOX‐resistance of breast cancer. Based on this, we detected that RES downregulated the expression of β‐catenin time‐ and dose‐dependently in MCF7/ADR cells. RES is a well‐known activator of SIRT1 which was reported to inhibit proliferation and migration through SIRT1‐mediated posttranslational modification of PI3K/Akt signaling in hepatocellular carcinoma cells.^27^ Another report revealed that SIRT1 promotes EMT in colorectal cancer by regulating Fra‐1.^28^ Also Bylesl V et al have reported that SIRT1 induces EMT by cooperating with EMT transcription factors and enhances prostate cancer cell migration and metastasis.^29^ Thus, whether SIRT1 is a EMT promoting factor or suppressor needs to be further explored. Chu et al^30^ reported that SIRT1 was upregulated in drug‐resistant cancer cell lines and patients’ tumor samples through increasing expression level of multidrug resistant protein 1, which was reminiscent of SIRT1’s promoting effect in the sensitivity of chemotherapy drugs. However, Deng et al^31^ found that the expression of SIRT1 was lower in prostate cancer, bladder cancer, ovarian cancer, and glioblastoma when compared with normal tissues. Thus, SIRT1 can function either as a promoter or a suppressor in chemotherapy resistance, depending on the tumor types, cellular background, or microenvironment. In our results, the protein level of SIRT1 in MCF7/ADR cells was lower than that in MCF7 cells, reminding SIRT1’s inhibitory effect on DOX‐resistance. Also, the protein level of SIRT1 was always upregulated under the treatment with DOX (Figure 5). Concordantly, our data suggested that RES activated expression of SIRT1 in MCF7/ADR cells in a time‐ and dose‐dependent manner (Figure 5). As EMT is associated with abnormal activation of canonical Wnt/PI3K/Akt pathway, which is correlated with the stabilization of β‐catenin.^32^, ^33^ We speculated that SIRT1 mediated β‐catenin signaling to reverse EMT and consequently inhibits cell migration in RES‐treated MCF7/ADR cells. In the results shown in Figure 6, we established an inverse link between SIRT1 and β‐catenin, and showed that up‐regulation of SIRT1 affected the expression level and stability of β‐catenin in MCF7/ADR cells.

By overexpressing SIRT1, phosphorylation level of β‐catenin decreased, as well as the ubiquitin‐mediated proteolysis of β‐catenin. Our data suggest that RES reverses DOX‐resistance through upregulating SIRT1 and then managing β‐catenin in MCF7/ADR cells.

## COMPETING INTERESTS

The authors declare that they have no competing interests.
