# Supplementary figures and images for: Resveratrol promotes sensitization to Doxorubicin by inhibiting epithelial‐mesenchymal transition and modulating SIRT1/β‐catenin signaling pathway in breast cancer
Source: Cancer Med. 2019 Jan 29;8(3):1246–57. doi: 10.1002/cam4.1993 (PMC6434195; doi:10.1002/cam4.1993)

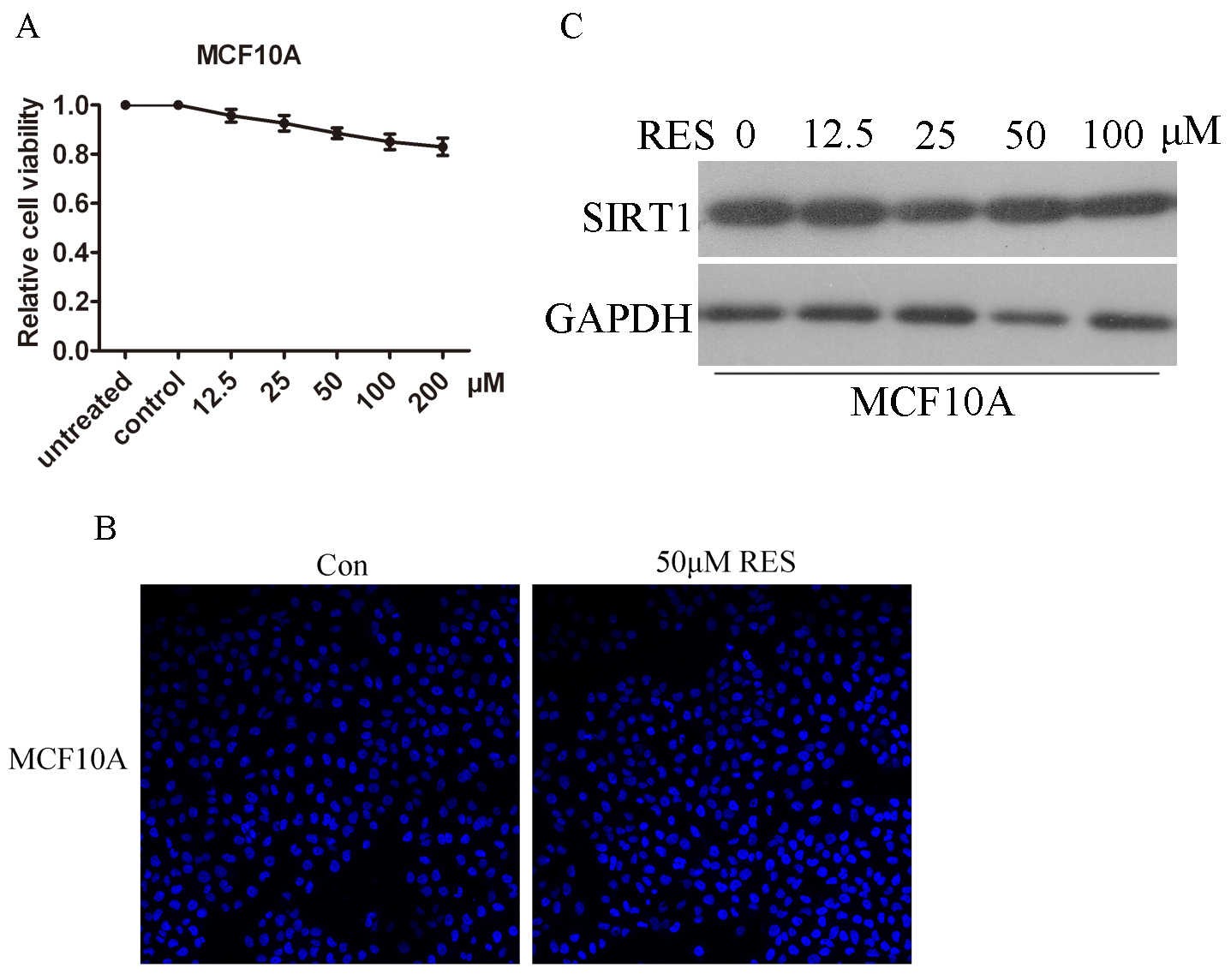

Supplement: Supplementary file 1 [file CAM4-8-1246-s001.tif]

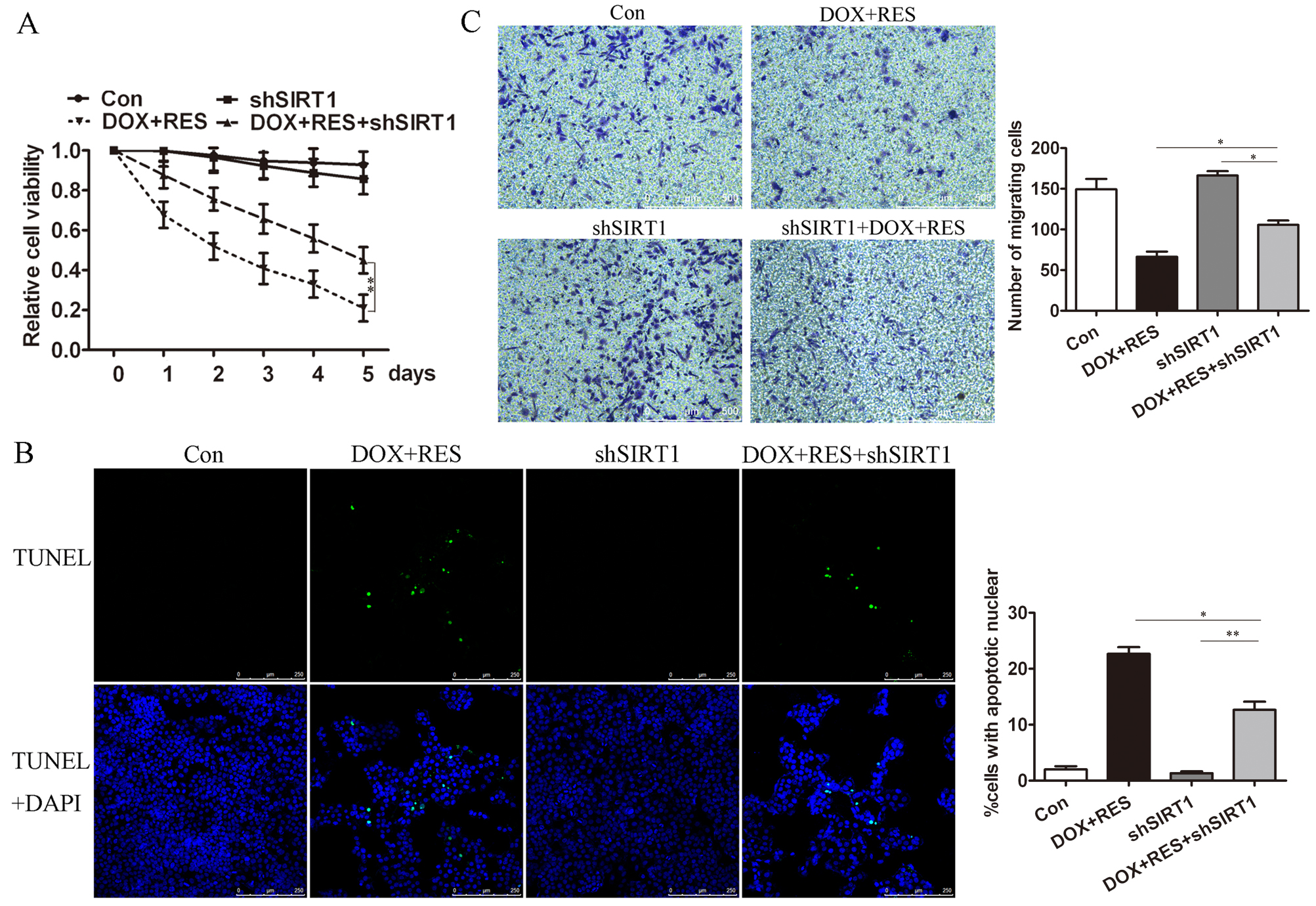

Supplement: Supplementary file 2 [file CAM4-8-1246-s002.tif]
